# Psychosocial Framework of Resilience: Navigating Needs and Adversities During the Pandemic, A Qualitative Exploration in the Indian Frontline Physicians

**DOI:** 10.3389/fpsyg.2021.622132

**Published:** 2021-03-16

**Authors:** Debanjan Banerjee, T. S. Sathyanarayana Rao, Roy Abraham Kallivayalil, Afzal Javed

**Affiliations:** ^1^Department of Psychiatry, National Institute of Mental Health and Neurosciences (NIMHANS), Bengaluru, India; ^2^Department of Psychiatry, JSS Medical College and Hospital, JSS Academy of Higher Education and Research, Mysuru, India; ^3^Department of Psychiatry, Pushpagiri Institute of Medical Sciences and Research Centre, Thiruvalla, India; ^4^Pakistan Psychiatric Research Centre, Fountain House Lahore, Islamabad, Pakistan; ^5^President, World Psychiatric Association (WPA), Geneva, Switzerland

**Keywords:** healthcare workers, physicians, COVID-19, resilience, psychosocial, challenges, frontline workers

## Abstract

**Introduction:**

Frontline healthcare workers (HCW) have faced significant plight during the ongoing Coronavirus disease 2019 (COVID-19) pandemic. Studies have shown their vulnerabilities to depression, anxiety disorders, post-traumatic stress, and insomnia. In a developing country like India, with a rising caseload, resource limitations, and stigma, the adversities faced by the physicians are more significant. We attempted to hear their “voices” to understand their adversities and conceptualize their resilience framework.

**Methods:**

A qualitative approach was used with a constructivist paradigm. After an initial pilot, a socio-demographically heterogeneous population of 172 physicians working in COVID-designated centers were purposively sampled from all over India. Following in-depth virtual interviews using a pre-formed semi-structured guide, the data was transcribed and translated verbatim. The interview was focused on their challenges, needs, and processes of coping and support. Charmaz’s grounded theory was used for analysis supplemented by NVivo 10 software.

**Results:**

Fear of infection, uncertainty, stigma, guilt, and social isolation emerged as the main challenges. Simultaneously, their “unmet needs” were flexible work policies, administrative measures for better medical protection, the sensitivity of media toward the image of HCW, effective risk communication for their health, and finally, social inclusion. Their resilience “framework” emerged as a process while navigating these adversities and consisted of three facets: forming a “resilient identity,” managing the resilience, and working through the socio-occupational distress. The role of mental well-being, social network, peer support, problem negotiation, and self-care emerged as the key coping strategies.

**Conclusion:**

The study findings support the global call for better psychosocial health and quality of life of the frontline HCWs. Their “unheard voices” explored in the study can anchor subsequent resilience-enhancing interventions and policies. Guidelines focusing on the psychological wellbeing of frontline HCWs need to be grounded in their unmet needs and lived experiences.

## Introduction

The unprecedented global crisis caused by the Coronavirus disease 2019 (COVID-19) pandemic has disproportionality affected many sections of the society. For obvious reasons, health care workers (HCW), especially those working on the frontline, are uniquely vulnerable to both the physiological and psychological offshoots of the outbreak (Chew et al., 2020; Greenberg et al., 2020; Vizheh et al., 2020). Even in the earlier Severe Acute Respiratory Syndrome (SARS), Middle East Respiratory Syndrome (MERS), and recently the Zika and Ebola outbreaks, the HCWs have faced overwhelming difficulties, chronic stress, high risk of infection and uncertainty, impaired quality of life and disturbed interpersonal relationships (Paladino et al., 2017; Simas et al., 2020; Xiao et al., 2020). Since the declaration of COVID-19 as a pandemic, multiple quantitative studies from various countries have explored the plight of the frontline physicians and reported increased rates of depression, anxiety, sleep disturbances, post-traumatic stress, and adjustment problems (Que et al., 2020; Spoorthy et al., 2020). With understaffing, rising caseload, and mental health-related stigma, the situation is even direr in a low and middle-income country (LMIC) like India, where the physician: patient ratio is 1:1,456 against the World Health Organization (WHO) recommendation of 1:1,000 (Paul and Bhatia, 2016). Considering the socio-cultural diversities and varied response to stressful situations, it is vital to understand the “unheard voices” of those fighting the pandemic at the upfront and qualitative approaches are better in that regard. Especially while navigating this adversity, it is important to appreciate their “processes of resilience” and strategies to improvise. According to Manning (2013), individuals who continue to manage hardships and flourish in personal and social lives are considered to be resilient; however, this concept of resilience has been highly contextualized based on the research settings and populations in whom it has been studied (Fletcher and Sarkar, 2013). Psychological resilience is the ability to emotionally cope with a crisis to return to the pre-critical state. It is said to exist when an individual uses “mental processes and behaviors in promoting personal assets and protecting self from the potential negative effects of stressors.” (De Terte and Stephens, 2014). The other way of looking at resilience is as a “psychological capital” that helps one stride through stressors and losses by the means of humor and hope (Pedro-Carroll and Jones, 2005). Emmy Werner, one of the first researchers who used the term resilience in 1970s after studying children in Hawaii, highlighted the need to understand resilience as a “fluid process” rather than a dichotomous construct that is built through constant interaction of an individual with his/her stressors and eventually helps in tiding over the adversity (Werner, 1971). Resilience research during biological disasters, maltreatment, abuse, violence, catastrophic life events, and poverty has focused on understanding the “processes” of resilience, so that it can be further enhanced through interventions (Grotberg, 1997; Werner, 2005). As resilience is considered as a dynamic interaction between individuals and the ongoing environment (Fletcher and Sarkar, 2013), we planned to explore the “lived experiences” of the frontline physicians, irrespective of their specialties, in terms of their challenges, unmet needs and further construct a “conceptual framework” of their psychological resilience during the ongoing crisis. Though HCW include many more specialties, it will be used interchangeably with physicians/doctors for the purpose of this study.

## Methodology

### Design and Sample

We adopted a qualitative design for the study with a social constructivist paradigm, especially as the objective was to gather “rich data” from the participants in terms of their lived experiences and explore the processes of their resilience. As opposed to the positivist approach in quantitative studies, social constructivism views knowledge to be constructed through constant interaction with others as human development is socially based (McKinley, 2015). In that way, social “realities” can be multiple based on the context, communication and interpretation all of which form the approach in qualitative research (which is based on social constructivism) (Walker, 2015). Qualitative methods have been shown to provide a substantial contribution to understanding the concept of resilience (Ungar, 2003). This is usually achieved through exploring lived experiences, phenomenological interpretation, understanding “minority voices,” constructing meaning of the “undefined” and member-checking of the results to establish trustworthiness (Ungar, 2003). We conceptualized resilience as a dynamic process that is difficult to be scaled or quantified and hence the approach to explore it needs to be “grounded” within the experiences of the population who use their resilience to navigate through the adverse situations. After being approved by the JSS-AHER Institutional Ethics Board, a semi-structured interview guide was designed based on detailed discussion among the researchers, existing literature related to the potential challenges faced by the frontline HCW, and clinical experience of the researchers (Box 1). It consisted of open-ended questions related to the experiences of the physicians while working in COVID-designated hospitals (as decided by the Government of India) (Government of India, 2020), the adversities that they have faced, their perceived needs while working, and how they attempted to overcome these hardships, including their sources of support, sense of control and narratives of the “process” of coping. The guide was supplemented by open-ended probes, prompts, and regular memo-writing to maintain the data trail. Only the salient questions have been mentioned in Box 1 for the sake of clarity. We theorized resilience as a flexible construct that lies on a dynamic continuum with inter-relationships between socio-cultural development and personal capacity building while exposed to stressful conditions, which can be altered and enhanced through various processes. The study used a theoretical and purposive sampling technique (Corbin and Strauss, 1990). The contact details were obtained through professional networks and directories of the national medical associations (Indian Psychiatric Society, Indian Medical Association), and snowballing was used to maximize sampling. We selected physicians of any specialty who were consistently working in a COVID-designated hospital (dealing with COVID-positive inpatients and outpatients) for at least 2 weeks. The time limit was arbitrary to exclude HCW, who are temporarily posted in COVID-wards on an *ad hoc* basis. Those who had been diagnosed with COVID-19 anytime in the last 6 months were excluded, which would alter their perceptions differently. All physicians were assessed by two independent psychiatrists (with a clinical consensus) before the interview to rule out any diagnosable mental health condition, in which case they would be excluded from the study. This was done as psychopathology could have been a potential confounding factor biasing the content of interviews, especially when it was related to the processes of coping during a stressful situation. Besides, a long, unbiased in-depth interview would not have been pragmatically and ethically possible with them. International Classification of Diseases (ICD)-10 was used for clinical diagnosis. A total of 28 participants were excluded in this way. Their symptoms may have been related to the professional stressors of the pandemic, however, the details of their diagnoses are not mentioned as they do not fall within the scope of this study. Irrespective of the participation status, they were provided required treatment by the psychiatrists involved.

**Box 1 Semi-structured interview guide used for the study. Difficulties during the pandemic:**

– How have the COVID-19 times been different for you (personally & professionally)?
– In what ways has the pandemic affected you and your loved ones?
– How do you feel about the ways you have been affected during this outbreak?
– What were the challenges that you faced as a physician during these times?
– How did you feel when you faced these challenges?
– How has the COVID-related lockdown impacted your clinical work, self-care and care for your family?
– What are the various factors that have led to these effects (that you mentioned above)?

Needs during the pandemic:

– How do you feel things could have been different during the outbreak (personal & professional font)?
– Based on the challenges mentioned above, what were your expectations from individuals/society/government?
– How did you feel about these expectations?
– What do you think your fellow healthcare workers felt during similar situations?
– How were those expectations met/not met? How did you feel about the same?

Coping during the pandemic:

– What were the positive things for you during the COVID-19 outbreak?
– How did these ‘positive aspects’ help you?
– How do you think your fellow healthcare workers fare during the pandemic? What factors may have helped them?
– Regarding the ‘challenges’ that you mentioned earlier, how did you deal with them?
– If you have overcome all/some of them, how did you do so?
– How has the pandemic changed you (as a healthcare worker and individual)? How do you feel about it?
– What would you suggest to others in similar situations of crisis?

Participants were sought based on varied ages, gender, all areas of India, practice settings, specialties, and socio-economic backgrounds. The contacts were initially mailed regarding the purpose, objectives, and nature of the study. Participants provided explicit informed consent, with whom virtual (Google Meet/Zoom/Skype) one-to-one detailed interviews were conducted over 1–2 sessions based on mutual convenience. The average session lasted 112 + −9.5 min. The open-ended questions of the interview guide were supplemented by various probing and supplementary queries to further obtain “rich” information, that forms the essence of qualitative research. However, the need and extent of probing varied between participants and were also based on the pragmatic feasibility of a virtual interview platform. All sessions were recorded with consent and conducted by the first three authors in English and Hindi. The initial pilot was done on 10 physicians, subsequent to which the interview guide was modified accordingly. The study was conducted between April-August 2020 and continued till the thematic saturation of data was obtained. To maintain anonymity and confidentiality, data sets were identified with a serial number/code and no names/identifiers were used. Furthermore, access to the participants’ interview recordings was strictly limited to the researchers. The participants were offered if they wanted to review the recordings or wished certain parts to be eliminated.

### Analysis

Charmaz’s grounded theory approach was used to analyze the data (Charmaz, 2006). Initially, all the interviews were transcribed and translated verbatim (with cross-translation) to ensure integrity. Subsequently, a frame-to-frame analysis was performed to obtain common contents or “codes,” which was the process of initial coding. Subsequently, focused coding and axial coding were performed to coalesce and condense codes into relevant themes and form a meaningful hierarchical structure between the resultant categories, themes, and codes, respectively. To enhance the level of clarity, causal references were looked for in the data and organized into a structure/process of relationships, which was important for exploring resilience. All steps of coding were done independently by the first two authors, who were certified in qualitative research. Through analysis, a constant comparison was made between the obtained themes and the actual “excerpts” back-and-forth to keep the results “grounded” in the data, along with syntheses of the themes based on rigorous discussion between the researchers. Though most of the coding was done manually as immersion into “rich data” is necessary for sound qualitative research, NVivo 10 software was used for assisting and organizing the analysis^1^.

The conceptual process of resilience was focused on during analysis. Thematic saturation was obtained with 162 participants, but 10 more were interviewed for super-saturation. Triangulation and respondent validation were further used to ensure study rigor (Krefting, 1991). The latter involved presenting the initial results to 60 participants from different ages, areas, and settings to discuss whether they truly “reflected” their perceptions and processes of coping during the crisis. Based on their subsequent inputs, further interviews were conducted, and interpretations were made accordingly. The entire analysis took 3 months to be complete.

## Results

The findings suggest how the physicians all over India working on the frontline faced the challenges and adversities during their service, their unmet needs and the “conceptual process” of their psychological resilience. Though we tried to keep the sampling as heterogeneous as possible, the participants were mostly married males practicing in Government set-ups of urban areas. The zonal representation in the sample was fairly equal, with more general physicians in the sample. Most physicians were young, in the age range of 20–30 years. The mean age and experience of the sample were 29.2 + −3.8 years and 16.7 + −4.2 years, respectively. The socio-demographic details of the participants are highlighted in Table 1.

**TABLE 1:** Socio-demographics of the participant physicians.

Attribute	Category	No. (*N* = 172)
Age (years)	20–30 30–40 40–50 >50	42 83 29 18
Gender	Male Female	110 62
Marital status	Single Married Divorced/separated	62 95 15
Region (India)	North South East West Central	32 68 30 32 20
Specialty	General physicians General Medicine/Pulmonologists Intensive care specialists Other specialties	74 52 25 21
Experience (years)	0–5 5–10 10–20 >20	25 34 92 21
Area of practice	Urban Semi-urban Rural	98 53 21
Set-up of practice	Government set-up Private organizations Private practice	110 47 15

Besides the challenges faced and the perceived needs, the results also reflect how the process of facing these hardships and the vulnerable state had helped their coping and resilience evolve through time. There were three facets to this:

• The resilient “identity or self” that was formed harnessing social support, rooted in morality, gratitude, and a sense of purpose (duties of a physician) that provided hope.
• The resilience “management” which occurred through regular dialogue with self and stress-management strategies that helped in problem-solving and negotiation with adversities. The sense of “togetherness” in the “physician community” enabled collectivism, which supplemented by their past training and stressful life-experiences helped them build resilience. Finally, the assumption of a “vulnerable or sick role” throughout the chronic stress of their challenges helped decrease expectations, promoted self-care, and reduced self-stigma.
• Working through the “distress” was facilitated by self-commitment and care (adequate sleep, diet, hobbies, small celebrations, festivities, etc.) that boosted self-confidence and positive lifestyle changes. They also drew their strength from their relationships, which was complemented by peer support, which proved valuable for their understanding, empathy, and validation. Telephonic sessions also helped them “work through” the adversities, and mental health was considered to be an important component of well-being. Finally, the participants agreed that facing the difficulties with a balanced and pragmatic approach was the only way to build resilience, as resilience and stress were bi-directional.

**FIGURE 1:**
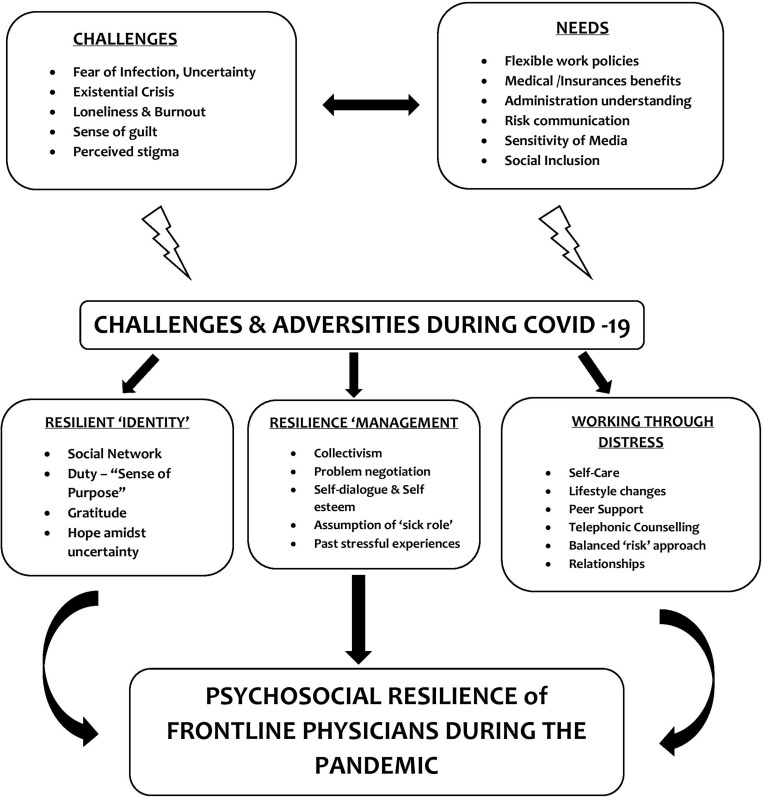
Psychosocial Resilience Framework of the Frontline Physicians during the COVID-19 pandemic as they navigate their needs and adversities: Through the processes of developing a resilient “identity,” managing the resilience resources and strategies of working through the “distress.”

The above-mentioned processes together formed the “conceptual framework” of the psychosocial resilience developed by our participant physicians while facing their challenges and adversities (Figure 1). This was grounded in their verbatim data obtained during the in-depth interviews.

Table 2 summarizes the key categories and themes, supported by the verbal excerpts from the participants. As the sample was large, we present only a few of the relevant excerpts in the results. The detailed participant-responses will be made available on reasonable request directed to the authors. Though there were no marked differences in the themes based on the gender, lady HCWs reported more challenges in work-life balance (“work from home” vs. “work for home”), especially those who were mothers. A greater proportion of them reported guilt of spreading the infection and social stigma compared to their male counterparts. While exploring resilience, we did not find any difference between the male and female HCWs.

**TABLE 2:** Categories, Themes of analysis and supporting verbal excerpts from the participants.

Categories	Themes (frequency)	Verbal Excerpts
Challenges	• Fear of infection and uncertainty (80%) • Existential crisis (65%) • Loneliness and burnout (69%) • Sense of Guilt (53%) • Perceived stigma (71%)	• “Each day is difficult. It’s like living with a constant sense of apprehension and guilt of infecting my family.” • *“I haven’t met my parents for months now. I stay separate to keep them safe. I have lost my colleague. Don’t know if I will lose them too*…*”* • *“People have started looking at me with “disgust”! It feels as if a doctor is always a carrier unless proven otherwise*…*”*
Unmet Needs	• Flexible work policies (88%) • Medical/Insurance benefits (70%) • Administrative understanding (60%) • Effective risk communication (43%) • Sensitivity of media (82%) • Social inclusion (90%)	• *“We are already understaffed. I haven’t got a single-day leave in the last 6 months. it can’t go on like this*…*”* • “The wards are not sanitized regularly. If the authority doesn’t organize, how will be managed such a caseload!” • *“Doctors are not immune. We work most closely with COVID patients. The degree of our risk estimates and shift rotations are mostly chaotic*… *that adds to our stress*…*”* • *“All that we need in such difficult times is some empathy. Many of my colleagues are being evicted from their apartments or looked down upon*…*”* • *“Doctors are being portrayed in a negative shade. this needs to stop! The popular media has a huge role to play in improving our status*…*”*
Processes of Resilience		
Resilient Identity	• Social network (55%) • Duty: “Sense of purpose” (67%) • Gratitude (42%) • Hope amidst uncertainty (49%)	• *“I am really thankful to my friends and family, who helped me move on, even from miles away*…*”* • *“I have seen the sufferings, deaths* and *grief myself, it makes me feel I can make a difference, save lives*…*”* • *“My duty and oath as a physician are my strengths, my hope*…*”*
Resilience management	• Collectivism (39%) • Problem negotiation (73%) • Dialogue with self and self-esteem (59%) • Assumption of “sick role” (52%) • Past stressful experiences (66%)	• *“I kept writing letters to myself*…*that was my stress-buster.”* • *“Now, I realize the importance of ICU duties and prolonged shifts. The training helps me gear up so much now*…*”* • *“There are multiple things at stake. I try my best to organize and prioritize at the end of the day*… *it helps me cope*…*”* • “While working in COVID-wards, we have to consider ourselves “vulnerable,” “potentially” sick: this stops too many expectations.” • *“I just keep telling myself, it’s a susceptible period, not to be too hard on myself*…*”*
Working through distress	• Self-care (73%) • Lifestyle changes (40%) • Peer support (84%) • Telephonic counseling (34%) • Balanced “risk” approach (47%) • Relationships (72%)	• *“Indulging in my hobbies and maintaining a schedule has helped me de-stress*…*”* • *“I felt my colleagues and co-workers understand my status best. I felt validated*…*”* • “Even weekly discussions with the counselor was fruitful. I felt there was an “audience” to my voice.” • *“Risk was inevitable since the pandemic started. You can’t avoid it, just try ways to minimize it*…*”* • *“You don’t cope till you face the risk. Face it in a pragmatic way that helps in the face of such distress*…*”*

## Discussion

### Challenges Faced by the Physicians

Our study identified the various key factors involved in building the process of resilience for the physicians working on the frontline. One of the prime challenges was perceived stigma and avoidance, which have been observed in healthcare since the beginning of the pandemic. Bagcchi (2020) mentioned about the condemning of more than 200 incidents of COVID-19 related attacks on health care workers by 13 medical and humanitarian organizations. Globally, the frontline HCWs have faced *“social ostracism,”* othering, discrimination, restrictions to public resources, and eviction from their apartments (Galbraith et al., 2020). Public fear and avoidance of them have been highlighted as an under-recognized form of stigmatization (Taylor et al., 2020). Media reports in India have occasionally portrayed doctors as “carriers of infections” and hence feared in the community (Bloomberg Quint, 2020). Since the beginning of the pandemic, xenophobic sentiments, and social prejudice were directed toward certain populations, especially the frontline workers. These have been compounded by misinformation related to the spread of infection, suggested remedies, and fear of “accessing healthcare facilities.” (Bhattacharya et al., 2020; Menon et al., 2020). With marked cultural diversities, pre-existing mental healthcare stigma, and varied societal beliefs, the perceptions toward HCW in India have been quite mixed. Such societal attitudes generate self-stigma in physicians, according to the Health-Stigma-Discrimination model, which facilitates internal hate, minimizes interactions, and causes social exclusion, further compounding isolation and burnout (Stangl et al., 2019). Guilt about transferring the infection to their loved ones, physical separation, and existential questions about the future of their families emerged as important themes in our study. This emotional insecurity also stemmed from a lack of perceived physical safety, as lack of adequate essential medical protective devices (PPE) has been reported as a consistent concern in the earlier studies from the United States (U.S.), China, and Saudi Arabia (Almaghrabi et al., 2020; Liu et al., 2020; Santarone et al., 2020). The constant fear of getting infected, decreased testing rates, and lack of leaves enhance the uncertainty, which along with “a helpless witness of daily suffering” in patients, creates “vicarious trauma” for the HCWs that impairs coping and accentuates chronic, complex trauma. Resource constraints, stigma related to mental illness even among physicians, and rising COVID-caseload in recent months potentially add to the burden.

### Needs

Based on the unprecedented global crisis created by the pandemic, the needs of the HCWs can be heterogeneous. Our participants welcomed the study as they felt that it provided an “audience” to their “unmet needs” and were quite expressive about the same during the interviews. A possible reason could be that the researchers were also physicians, which could have acted as a “peer support.” Flexible working hours, insurance coverage, and adequate medical safety are concerns that have been resonated worldwide (Taylor et al., 2020; Vindrola-Padros et al., 2020), but assume renewed significance in developing countries. One of the first studies done on Indian physicians during Lockdown highlighted the need for administrative assurance, financial security, recognition and societal understanding as important factors for altruistic coping (Chatterjee et al., 2020). Recognition and support of staff in healthcare are vital factors for confidence, motivation, sense of achievement, and occupational security (Abu Sultan et al., 2018). Interestingly, social inclusion, timely risk communication by the infection control committee to the HCWs, and the presence of “medical trust” in the system were the predominant needs reported in our study. With a widening treatment gap and lack of primary – tertiary collaboration, this can be a significant concern in India. The benefits of periodic mental health screenings, digital peer support groups, and counseling sessions have demonstrated benefits in other pandemic-struck countries like China and Italy (Di Tella et al., 2020; Liu et al., 2020). Our participant HCWs perceived that their “emotional needs” lacked ears and, with the added stigma, further isolated them from the mainstream. Such social exclusion has been shown to increase apprehension and uncertainty, which can potentially increase experiential avoidance, enhancing stress (Seçer et al., 2020). As pointed out by Banerjee et al. (2020) in the systematic and advocacy review of the Indian Psychiatric Society (IPS) related to COVID-19 and psychological well-being, the need for safeguarding physical, financial security and psychosocial healthcare of frontline physicians are supposed to serve as important parameters in the fight against the pandemic. Though the guidelines of the Ministry of Health and Family Welfare (MoHFw), Government of India (GOI) mention the “selfless service” and critical roles of frontline HCW including nurses in the community and call upon for national stigma-mitigating techniques, our study indicates that the practical implementation of such guidelines is still a way to go. The available guidelines for psychosocial wellbeing during emergencies focus more on categorical definitions of distress and fail to tap into the resilience of the frontline workers. A study by San Juan et al. (2020) contrasted guidelines with lived experiences of practicing HCWs in United Kingdom and reported that understaffing, mental exhaustion and busy schedules often prevented them from accessing the available interventions. Future research can focus on exploring the gaps in the current guidelines when compared to the unmet needs and perceptions of HCWs in India during the pandemic crisis.

### Resilience as a Process

Most of our participants mentioned resilience as a continuum developed through experiencing and facing an unprecedented crisis, aided by social support and past encounters with stress. Traditionally, schools of thought have debated on the static versus dynamic views of resilience, which pave the way for resilience-building strategies and interventions (Werner, 2005; Fletcher and Sarkar, 2013). Our findings grounded in the experiences of our physicians support the “learned resilience” hypothesis (Ryff, 2014), conceptualizing the framework through a “resilience identity,” managing the gradual development of resilience and working through the ongoing distress. Hence it is a process that can be intervened with therapeutic strategies, adaptive coping, resilience training, stress counseling, etc. It has also been related to secondary trauma faced by the HCWs, which can cause biopsychosocial impairment and decisional inefficacy in physicians during the pandemic (Vagni et al., 2020). Based on the conceptualization of resilience in our study as mentioned before, our results suggested that the consistent living through hardships and adversities of the COVID-19 crisis with responsible risk-taking helped pave the way for problem-solving, personal efficiency, and coping in the physicians.

An overarching theme in our study was a physician’s duty and moral obligation to serve during crisis situation, which provided the physicians with a “moral sense of purpose” and formed the basis of a resilient self. HCWs derived hope and gratitude from the same with further help from their social connections. This engagement process has been theorized during COVID-19 to help combat loneliness and isolation, turning them into resilient and self-subserving “solitude” (Banerjee and Rai, 2020). Further, our participants also reported the timeliness of activating these social supports in order to prevent reaching the breaking point. Based on their prior experiences, reframing of hardships was a vital factor. Few studies of resilience and resources in HCWs done earlier have identified work as “personal gratification” and “doctor’s duty as a resilience among challenges” in HCWs (Ardebili et al., 2020; Liu et al., 2020). The latter study though done on a much smaller sample, was one of the first to identify that a physician’s “training, oaths, and values” were related to coping and resilience in such crisis situations. Social constructivism in qualitative research can have various approaches. Nyashanu et al. (2020) used interpretive phenomenological analysis (IPA) to study the lived experiences of 40 frontline HCWs in Midlands, United Kingdom. They worked mainly in the private care homes and domiciliary care agencies. Death of colleagues, fear of infecting others, unreliable testing and shortage of staff were reported as important concerns in the study. The participants felt that poor preparedness for the pandemic crisis had affected their coping adversely. Psychological preparedness as well as advance public health measures have been suggested as vital strategies to deal with the pandemic burden in a socio-culturally heterogenous and populous nation like India (Banerjee, 2020). The HCWs in this study also added to this by mentioning that public understanding and social support during the initial phase of the COVID-19 crisis had boosted the process of their resilience. A community-based psychosocial toolkit based on the Zika virus model that was proposed to deal with the pandemic burden in India also includes resilience building among frontline workers through sense of purpose, social support and social cohesion at all levels of healthcare (Banerjee and Nair, 2020).

The next attribute was managing the *“praxis of resilience”* through an enhanced sense of self-esteem and maintaining it through self-dialogue. Socio-cultural diversities existed in our sample interviewed from various parts of India, and the ways, as well as processes of coping varied but there, was a commonality in “problem negotiation” (confronting and reframing the problem areas). Based on Cognitive-Behavioral principles, this is considered as a healthy problem-solving approach that involves perceived self-efficacy (Brown et al., 2012). Further, many HCWs assumed a “sick role” considering themselves exposed to the infection, which helped them reduce personal expectations and perceived guilt. The sick role has shown to be beneficial during the chronic crisis as per sociological theories and help attributes the impaired performance and socio-occupational shortcomings of sick or vulnerable individuals to the ongoing adverse situations, which creates a “shielding” from enhanced roles and responsibilities during a crisis situation (Shilling, 2002). Hope and adaptation to a different lifestyle were reported by more than half of our participants as coping mechanisms which they though would fetch them more experience during the post-pandemic aftermath. “New normalization” and using gained experiences to adapt to the pandemic stress have also been reported in a qualitative study from Iran where 97 HCWs including emergency services, physicians, nurses, pharmacists, laboratory personnel, radiology technicians, etc. reported change in personal lifestyle, new experiences in the pandemic era, negative emotions, learning to deal with them and finally need for mental health interventions as the major themes of their lived experiences while working during the outbreak (Ardebili et al., 2020).

Also, based on each of their training and experiences, they developed a “resilience model” consisting of mutual support among the peers, empathy, and positivism that generated hope in spite of adversities. Earlier studies on lived experiences during pandemic reported positivism and collectivism as powerful coping strategies that also help in the reduction of stigma. Xenophobia has been a growing concern during the pandemic, compounded by misinformation, which has been shown to increase fear of infection and can be potentially mitigated by collectivistic attitudes and personal growth (Ahuja et al., 2020). Xenophobic attitudes and social stigma was experienced by a greater proportion of lady HCWs in our study. This has been resonated earlier as well, where “amplification of social inequalities, paternalistic discourses and professional overshadowing of personal lives” were prominent among female NHS frontline health workers (Yarrow and Pagan, 2020).

Finally, our participants agreed on the “gray line” of calculated risk-taking as part of occupational hazards with the strategic precautions, which boosted medical and emotional security. Most of them admitted that when “escape from stress” is impossible, facing it helps in the process of coping, whereas avoidance makes it chronic. The ongoing adversities of working in a pandemic situation helped them sustain personally and professionally. They discussed retaining a positive image while facing vulnerabilities and stressors on a pre-planned support system, through digital connectedness with peer groups and enjoying their hobbies and small celebrations. Studies have shown that groups with similar occupations can emphasize better during the crisis, which was resonated by the strength derived by our physicians from peer-support. “The risk with reason” approach helped our participants “work through the distress” aided by hobbies, support, spirituality or positivism, and the HCWs were quite open to discussion of how to focus on diet, nutrition, sleep, and lifestyle through generic measures, aided in resilience. Positive risk-taking has been related to risk-perception during infectious disease outbreaks, which in turn influence psychological wellbeing. Studies from China and Italy have shown how health risk perception can be influenced by empathy, self-efficacy and positive imagination (Commodari et al., 2020; Ding et al., 2020). The HCWs participants in our study also mentioned that a “careful balance between risk adaptation and medical safety measures” helped them face the prolonged stress of work during the pandemic and they faced reduced personal susceptibility to the infection. Self-care and management have been considered as a dynamic interaction between an individual and his/her stressful circumstances that determine overall health and well-being. The well-known concept of micro-resilience is also related to self-care, self-esteem, and internal locus of control (Ryff, 2014). These factors can help in fostering resilience through the lifespan, which forms the “psychological capital” during a crisis. Our study also revealed that the simple measures of telephonic counseling provided validation and an “emotional audience,” which went a long way for emotional support. This has been the basis for the telecare model in china for HCWs in hospitals of Hubei province, where the pandemic first appeared. In short, the overall process of resilience was highly contextualized and related to the socio-occupational environment, but irrespective of the personal strategies used, the results help in conceptualizing a common ground in the “resilience-framework” of physicians during the ongoing outbreak. Such focused social support and understanding of the distress faced by HCWs during crisis times can help reduce social stigma and improve social connections. This has been termed as an “epidemic of empathy” that has the potential to bring together science and humanism that might be beneficial even after cessation of the pandemic (Barello and Graffigna, 2020). As discussed before, empathy, optimism and self-efficacy can also improve personal health-risk perception, which is vital for psychological resilience during pandemics (Commodari et al., 2020).

The study has the usual limitations of qualitative work, including generalizability and subjectivity. Besides, we only included physicians in the sample, while HCW also consists of nurses, para-medical staff, and other allied professionals. However, the study sample was large and heterogenous in socio-demographics, from all parts of India. Also, the rich data of the lived experiences of the physicians and rigorous analysis are the added strengths of the study. Besides this, we had to exclude some participants as they were diagnosed to be psychiatrically ill by independent psychiatrists prior to the commencement of the study. Only clinical interview was used though diagnosis was established through a consensus. The authors agree that some of these mental health issues could have been contributed by the psychosocial stressors of working during COVID-19. However, the objective of the present study was to explore resilience framework and coping in HCWs and pre-existing psychopathology would have colored their subjective perceptions during the pandemic, which form the main data of this qualitative study.

## Conclusion

The psychosocial well-being of the physicians strengthens the healthcare infrastructure, which is vital for any country. With growing caseload, increased work-burden, and resource constraints, the quality of life of HCW assumes exaggerated importance in developing countries like India. To the best of our knowledge, this is the first study from any LMIC to explore the “voices” of those directly working with COVID-19 patients and conceptualize their processes of resilience. Santarone et al. (2020) highlighted the importance of incorporating the needs and perspectives of HCW into resilience-building strategies that can involve mental health screenings, peer support, sensitive workplace infrastructure, and social security. Stigma-mitigating strategies need to be a collective responsibility for all levels of stakeholders, including sensitive reporting by the media. Bhattacharya et al. (2020) while discussing the consequences of social stigma in India, mentions the “dual burden” of the pandemic and prejudice in HCWs, suggesting the need to amplify their voices for psychosocial management and administrative policymaking. The “resilience framework” derived in the study can be integrated into digital psychotherapeutic interventions involving cognitive-behavioral, interpersonal and humanistic principles. The post-pandemic aftermath is uncertain, and various public health agencies have globally called for the safety and resilience-building of the frontline HCW (Banerjee et al., 2020; Galbraith et al., 2020; Xiao et al., 2020). This study provides a small step toward that “call” and obviously warrants further systematic, population-based, and mixed-method research into the emotional and psychosocial well-being of the HCW, their mental health issues, hardships at work, and finally the ways of coping, which can shape tailored interventions and legislations. There is also an urgent need to tailor the existing guidelines for the psychosocial wellbeing of the frontline HCWs based on their unmet needs and lived experiences. This much-needed approach can potentially anchor the ongoing fight against the pandemic and help preparedness for such futuristic crises.

## Data Availability Statement

The raw data supporting the conclusions of this article will be made available by the authors, on reasonable request.

## Ethics Statement

The studies involving human participants were reviewed and approved by the JSS University, Mysore, India. The patients/participants provided their written informed consent to participate in this study.

## Author Contributions

DB and AJ share the corresponding authorship. DB, TS, and AJ were responsible for the data collection and curation. DB and TS were involved in data analysis. DB and RK worte the first draft of the manuscript. All authors had conceptualized the study and design, had full access to the data and take responsibility for data integrity and analysis, responsible for the reviewing, editing, and final approval of the manuscript.

## Conflict of Interest

The authors declare that the research was conducted in the absence of any commercial or financial relationships that could be construed as a potential conflict of interest.

## Footnotes

1 www.qsrinternational.com
